# DateLife: Leveraging Databases and Analytical Tools to Reveal the Dated Tree of Life

**DOI:** 10.1093/sysbio/syae015

**Published:** 2024-03-20

**Authors:** Luna L Sánchez Reyes, Emily Jane McTavish, Brian O’Meara

**Affiliations:** Department of Life and Environmental Sciences, University of California, Merced, CA 95343, USA; Department of Ecology and Evolutionary Biology, University of Tennessee, Knoxville, 446 Hesler Biology Building, Knoxville, TN 37996, USA; Department of Life and Environmental Sciences, University of California, Merced, CA 95343, USA; Department of Ecology and Evolutionary Biology, University of Tennessee, Knoxville, 446 Hesler Biology Building, Knoxville, TN 37996, USA

**Keywords:** Ages, calibrations, congruification, dating, divergence times, open science, phylogeny, scaling, secondary calibrations, supertree, tree

## Abstract

Chronograms—phylogenies with branch lengths proportional to time—represent key data on timing of evolutionary events, allowing us to study natural processes in many areas of biological research. Chronograms also provide valuable information that can be used for education, science communication, and conservation policy decisions. Yet, achieving a high-quality reconstruction of a chronogram is a difficult and resource-consuming task. Here we present DateLife, a phylogenetic software implemented as an R package and an R Shiny web application available at www.datelife.org, that provides services for efficient and easy discovery, summary, reuse, and reanalysis of node age data mined from a curated database of expert, peer-reviewed, and openly available chronograms. The main DateLife workflow starts with one or more scientific taxon names provided by a user. Names are processed and standardized to a unified taxonomy, allowing DateLife to run a name match across its local chronogram database that is curated from Open Tree of Life’s phylogenetic repository, and extract all chronograms that contain at least two queried taxon names, along with their metadata. Finally, node ages from matching chronograms are mapped using the congruification algorithm to corresponding nodes on a tree topology, either extracted from Open Tree of Life’s synthetic phylogeny or one provided by the user. Congruified node ages are used as secondary calibrations to date the chosen topology, with or without initial branch lengths, using different phylogenetic dating methods such as BLADJ, treePL, PATHd8, and MrBayes. We performed a cross-validation test to compare node ages resulting from a DateLife analysis (i.e, phylogenetic dating using secondary calibrations) to those from the original chronograms (i.e, obtained with primary calibrations), and found that DateLife’s node age estimates are consistent with the age estimates from the original chronograms, with the largest variation in ages occurring around topologically deeper nodes. Because the results from any software for scientific analysis can only be as good as the data used as input, we highlight the importance of considering the results of a DateLife analysis in the context of the input chronograms. DateLife can help to increase awareness of the existing disparities among alternative hypotheses of dates for the same diversification events, and to support exploration of the effect of alternative chronogram hypotheses on downstream analyses, providing a framework for a more informed interpretation of evolutionary results.

Chronograms—phylogenies with branch lengths proportional to time—provide key data on evolutionary time frame for the study of natural processes in many areas of biological research, such as comparative analysis (Freckleton et al. 2002; Harvey et al. 1991), developmental biology (Delsuc et al. 2018; Laubichler & Maienschein 2009), conservation biology and ecology (Felsenstein 1985; Webb 2000), historical biogeography (Posadas et al. 2006), and species diversification (Magallon & Sanderson 2001; Morlon 2014).

Building a chronogram is not an easy task. It requires obtaining and curating a homology hypothesis to construct a phylogeny, selecting and placing appropriate calibrations on the phylogeny using independent age data points from the fossil record or other dated events, and inferring a full dated tree. All of this entails specialized biological training, taxonomic domain knowledge, and a significant amount of research time, computational resources and funding.

Here, we present the DateLife project which has the main goal of extracting and exposing age data from published chronograms, making age data readily accessible to a wider community for reuse and reanalysis in research, teaching, science communication, and conservation policy. DateLife’s core software application is available as an R package (Sanchez-Reyes et al. 2022), and as an online Rshiny interactive website at www.datelife.org. It features key elements for scientific reproducibility, such as a curated, versioned, open and fully public chronogram database (McTavish et al. 2015) that stores data in a computer-readable format (Vos et al. 2012); automated and programmatic ways of accessing and downloading the data, also in a computer-readable format (Stoltzfus et al. 2013); and methods to summarize and compare the data.

## Description

DateLife’s core software applications are implemented in the R package datelife, and relies on functionalities from other biological R packages: ape (Paradis et al. 2004), bold (Chamberlain 2018), geiger (Pennell et al. 2014), msa (Bodenhofer et al. 2015), paleotree (Bapst 2012), phyloch (Heibl 2008), phylocomr (Ooms & Chamberlain 2018), phytools (Revell 2012), rotl (Michonneau et al. 2016), and taxize (Chamberlain 2018; Chamberlain & Szöcs 2013). There are 3 main steps to the DateLife workflow: (1) creating a search query, (2) searching a database, and (3) summarizing results from the search.

### Creating a Search Query

DateLife starts by processing an input consisting of the scientific name of at least one taxon. Multiple input names can be provided as a comma separated character string or as tip labels on a tree. If the input is a tree, it can be provided as a classic newick character string (Archie et al. 1986), or as a “phylo” R object (Paradis et al. 2004). The input tree is not required to have branch lengths, and its topology is used in the summary steps described in the next section.

DateLife processes input scientific names using a Taxonomic Name Resolution Service (TNRS), which increases the probability of correctly finding the queried taxon names in the chronogram database. TNRS detects, corrects, and standardizes name misspellings and typos, variant spellings and authorities, and nomenclatural synonyms to a single taxonomic standard (Boyle et al. 2013). TNRS also allows to correctly choose between homonyms, by considering other taxa provided as input to infer the taxonomic context of the homonym. DateLife implements TNRS using the Open Tree of Life (OpenTree) unified Taxonomy (Open Tree Of Life et al. 2016; Rees, & Cranston 2017) as standard, storing taxonomic identification numbers (OTT ids) for further processing and analysis. Other taxonomies currently supported by DateLife are the National Center of Biotechnology Information (NCBI) taxonomic database (Schoch et al. 2020), the Global Biodiversity Information Facility (GBIF) taxonomic backbone (GBIF Secretariat 2022), and the Interim Register of Marine and Non-marine Genera (IRMNG) database (Rees, & Cranston 2017).

Besides binomial species names, DateLife accepts scientific names from any inclusive taxonomic group (e.g., genus, family, and tribe), as well as subspecific taxonomic variants (e.g., subspecies, variants, and strains). If a taxon name belongs to an inclusive taxonomic group, DateLife has 2 alternative behaviors defined by the “get species from taxon” flag. If the flag is active, DateLife retrieves all species names within a taxonomic group provided, from a standard taxonomy of choice, and adds them to the search query. In this case, subspecific variants are excluded. If the flag is inactive, DateLife excludes inclusive taxon names from the search query, and species and subspecific variant names are processed as provided by the user. The processed taxon names are saved as an R object of a newly defined class, datelifeQuery, that is used in the following steps. This object contains the input names standardized to a taxonomy of choice (OTT by default), the corresponding OTT id numbers, and the topology of an input tree, if one was provided.

### Searching a Chronogram Database

At the time of writing of this manuscript (Mar 08, 2024), DateLife’s chronogram database latest version consist of 253 chronograms published in 187 studies, encompassing 99,474 species. It is curated from OpenTree’s phylogenetic database, the Phylesystem, an open database of expert and peer-reviewed phylogenetic knowledge with rich metadata and a wide taxonomic scope (McTavish et al. 2015). We expect DateLife’s database to largely overlap with OpenTree’s phylogenetic database taxonomic coverage, where Chordata and Embryophyta are nearly fully sampled. In contrast, Bacteria, Fungi, Nematoda, and Insecta, currently present a large gap between the number of named species and what has phylogenetic information in OpenTree’s synthetic tree. It is likely that users working with the former groups will get results from a DateLife analysis. If none of the user’s species are found, the software will indicate the lack of age data for the queried taxa in the database.

A unique feature of the Phylesystem is that any user can add new published, state-of-the-art chronograms any time, through OpenTree’s curator application (https://tree.opentreeoflife.org/curator). Relying on an open source database permits an automatic and reproducible assembly of DateLife’s chronogram database, which is stored and navigable as an R data object within the datelife R package. As chronograms are added to Phylesystem, they can be incorporated into the chronogram database within the datelife R package, by manually triggering an update. The updated datelife database is assigned a new version number, followed by a package release on CRAN. We encourage users to submit published chronograms to OpenTree’s phylogenetic database, so that their taxon of interest can be included in future DateLife searches. Users can directly run datelife functions to trigger an update of their local chronogram database, to incorporate any new chronograms to their DateLife analysis before a datelife database update is released on CRAN.

A DateLife search is implemented by matching processed taxon names provided by the user to tip labels in the chronogram database. Chronograms with at least 2 matching taxon names on their tip labels are identified and pruned down to preserve only the matched taxa. These matching pruned chronograms are referred to as source chronograms. Total distance in units of million years (myr) between taxon pairs within each source chronogram are stored as a patristic distance matrix. The matrix format speeds up extraction of pairwise taxon ages of any queried taxa, as opposed to searching the ancestor node of a pair of taxa in a “phylo” object or newick string. Finally, the patristic matrices are associated to the study citation where the original chronogram was published, and stored as an R object of the newly defined class datelifeResult.

### Summarizing Search Results

Summary information is extracted from the datelifeResult object to inform decisions for subsequent steps in the analysis workflow. Basic summary information available to the user includes:

1. The matching pruned chronograms as newick strings or “phylo” objects.
2. The ages of the root of all source chronograms. These ages can correspond to the age of the most recent common ancestor (mrca) of the user’s group of interest if the source chronograms have all taxa belonging to the group. If not, the root corresponds to the mrca of a subgroup within the group of interest.
3. Study citations where original chronograms were published.
4. A report of input taxon names matches across source chronograms.
5. The source chronogram(s) with the most input taxon names.
6. Various single summary chronograms resulting from summarizing age data, generated using the methodology described next.

### Choosing a Topology

DateLife requires a tree topology to summarize age data upon. We recommend that users provide as input a tree topology from the literature, or one of their own making. If no topology is provided, DateLife automatically extracts one from the OpenTree synthetic tree, a phylogeny currently encompassing 2.3 million taxa across all life, assembled from 1,239 published phylogenetic trees and OpenTree’s unified Taxonomy, OTT (Open Tree Of Life et al. 2019). Alternatively, DateLife can combine topologies from source chronograms using a supertree approach (Criscuolo et al. 2006). To do this, DateLife first identifies the source chronograms that form a grove, roughly, a sufficiently overlapping set of taxa between trees, by implementing definition 2.8 for n-overlap from Ané et al. (2009). If the source chronograms do not form a grove, the supertree reconstruction will fail. In rare cases, a group of trees can have multiple groves. By default, DateLife chooses the grove with the most taxa; however, the “criterion = trees” flag allows the user to choose the grove with the most trees instead. The result is a single summary (i.e., supertree) topology, that combines topologies from source chronograms in a grove.

### Applying Secondary Calibrations

Once a topology is chosen, DateLife applies the congruification method (Eastman et al. 2013) that find nodes belonging to the same clade across source chronograms, and then extracts the corresponding node ages from patristic distance matrices stored as a datelifeResult object. Note that by definition, these matrices store total distance (time from tip to tip), assuming that the terminal taxa are coeval and occur at the present. Hence, node ages correspond to half the values stored in the datelifeResult matrices. A table of congruified node ages that can be used as calibrations for a dating analysis is stored as a congruifiedCalibrations object.

For each congruent node, the pairwise distances that traverse that node are summarized into a single summary matrix using classic summary statistics (i.e., mean, median, minimum, and maximum ages), and the Supermatrix Distance Method (SDM; Criscuolo et al. 2006), which deforms patristic distance matrices by minimizing variance and then averaging them. These single summary taxon pair age matrices are stored as summarized calibrations that can be used as secondary calibrations to date a tree topology—with or without initial branch lengths, using phylogenetic dating methods currently supported within DateLife: BLADJ (Webb et al. 2008; Webb & Donoghue 2005), MrBayes (Huelsenbeck & Ronquist 2001; Ronquist & Huelsenbeck 2003), PATHd8 (Britton et al. 2007), and treePL (Smith & O’Meara 2012).

### Dating a Tree Topology

#### Dating a tree without branch lengths.—

To date a tree topology when initial branch lengths are unavailable, DateLife implements the Branch Length Adjuster (BLADJ) algorithm (Webb et al. 2008; Webb & Donoghue 2005), which only requires a tree topology with no branch lengths and at least two node ages to use as calibrations, one for the tree root and one for any internal node of the topology. The BLADJ algorithm fixes ages for nodes with calibration data upon the given tree topology. Then, it assigns ages to nodes with no available age information by distributing time evenly between calibrated nodes, minimizing age variance in the resulting chronogram. This approach has proven useful for ecological analyses that require a phylogenetic time context (Webb et al. 2008). When there is conflict between ages of calibrated nodes, BLADJ ignores node ages that are older than the age of a parent node. The BLADJ algorithm requires a root age to run. Users can provide an appropriate root age estimate of their own or one obtained from the literature. If a root age is not provided and there is no information on the age of the root in the chronogram database, DateLife chooses a random age for the root, so that a dated tree topology can be generated with BLADJ. In this case, DateLife will provide a conspicuous warning message, so that users are aware that the root of the chronogram was chosen at random because there was no information available for it in the chronogram database, along with suggestions on how the user can find and provide an appropriate age for the root of the initial topology.

An alternative to BLADJ to date tree topologies in the absence of initial branch lengths that is common practice in the literature is to use a birth-death model to draw branch lengths (Jetz et al. 2012; Rabosky et al. 2018; Smith 2018). In addition to the initial tree topology and nodes with age data, these methods require initial values of speciation and extinction rate parameters provided by the user. DateLife implements this approach with MrBayes (Huelsenbeck & Ronquist 2001; Ronquist & Huelsenbeck 2003), using nodes with published age data as calibration priors on nodes of a tree topology with no branch lengths, a simple birth-death model with speciation and extinction rate parameters that are provided by the user, and no genetic data. However, BLADJ is the default option in DateLife, as it does not require any information on diversification rates for the phylogenetic sample to draw from a branch length distribution.

#### Dating a tree with branch lengths.—

Relative branch lengths can provide key information for phylogenetic dating, specifically for nodes without any calibration data available. While using initial branch length data is the golden standard for phylogenetic dating analyses, obtaining such information from scratch is not an easy task: it requires obtaining primary data, assembling and curating a homology (orthology) hypothesis, and choosing and implementing a method for phylogenetic inference. DateLife implements a workflow to streamline this process by applying open data from the Barcode of Life Data System, BOLD (Ratnasingham & Hebert 2007) to obtain genetic markers for input taxa. By default, BOLD genetic sequences are aligned with MUSCLE (Edgar 2004) using functions from the msa R package (Bodenhofer et al. 2015). Alternatively, sequences can be aligned with MAFFT (Katoh et al. 2009), using functions from the ape R package (Paradis et al. 2004). The BOLD sequence alignment is then used to obtain initial branch lengths with the accelerated transformation (ACCTRAN) parsimony algorithm, which resolves ambiguous character optimization by assigning changes along branches of the tree as close to the root as possible (Agnarsson & Miller 2008), resulting in older internal nodes as compared to other parsimony algorithms (Forest et al. 2005). The parsimony branch lengths are then optimized using maximum likelihood, given the alignment, the topology and a simple Jukes–Cantor model, producing a BOLD tree with branch lengths proportional to expected number of substitutions per site. Both parsimony and ML optimizations are done with functions from the phangorn package (Schliep 2011). Due to the computing load it requires, the BOLD workflow is currently only supported through DateLife’s R package. It is not yet available through the web application.

Phylogenetic dating methods supported in DateLife that incorporate branch length information from the input topology in combination with the secondary calibrations include: PATHd8, a non-clock, rate-smoothing method to date trees (Britton et al. 2007); treePL (Smith & O’Meara 2012), a semi-parametric, rate-smoothing, penalized likelihood dating method (Sanderson 2002); and MrBayes (Huelsenbeck & Ronquist 2001; Ronquist & Huelsenbeck 2003), a Bayesian inference program implementing Markov chain Monte-Carlo (MCMC) methods to estimate a posterior distribution of model parameters.

### Visualizing Results

Finally, users can save all source and summary chronograms in formats allowing for reuse and reanalysis, such as newick and the R “phylo” format. Input and summary chronograms can be visualized and compared graphically, and users can construct their own graphs using DateLife’s chronogram plot generation functions available from the R package datelifeplot (Sanchez-Reyes et al. 2022).

## Benchmark

**Figure 1:**
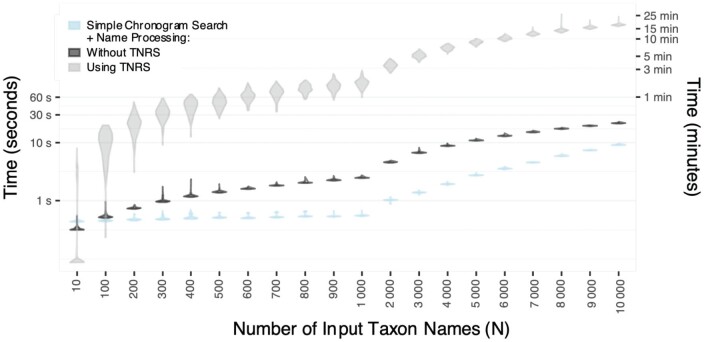
DateLife’s benchmarking results showing computation time used for taxon name processing and search across datelife’s chronogram database, as a function of number of input taxon names (N). For each N = {10, 100, 200, …, 1 000, … , 9 000, 10 000}, we randomly sampled N species names from the class Aves, a hundred times, and then performed a datelife search processing the input names using the Taxon Names Resolution Service (TNRS; light gray), and without processing input names (dark gray). For comparison, we performed a chronogram search using names that have been pre-processed with TNRS.

R package datelife code speed was tested on an Apple iMac with one 3.4 GHz Intel Core i5 processor. We registered variation in computing time of query processing and search through the database relative to number of queried taxon names. Query processing time increases roughly linearly with number of input taxon names, and increases considerably if Taxonomic Name Resolution Service (TNRS) is activated. Up to 10,000 names can be processed and searched in less than 30 min with the most time-consuming settings. Once names have been processed as described in Methods, a name search through the chronogram database can be performed in less than a minute, even with a very large number of taxon names (Fig. 1).

datelife’s code performance was evaluated with a set of unit tests designed and implemented with the R package testthat (R Core Team 2018) that were run both locally with the devtools package (R Core Team 2018), and on a public server using the continuous integration tool of GitHub actions (https://docs.github.com/en/actions). At present, unit tests cover more than 40% of datelife’s code (https://codecov.io/gh/phylotastic/datelife). Unit testing helps identify potential issues as code is updated or, more critically, as services code relies upon may change.

## Case Studies

**Figure 2:**
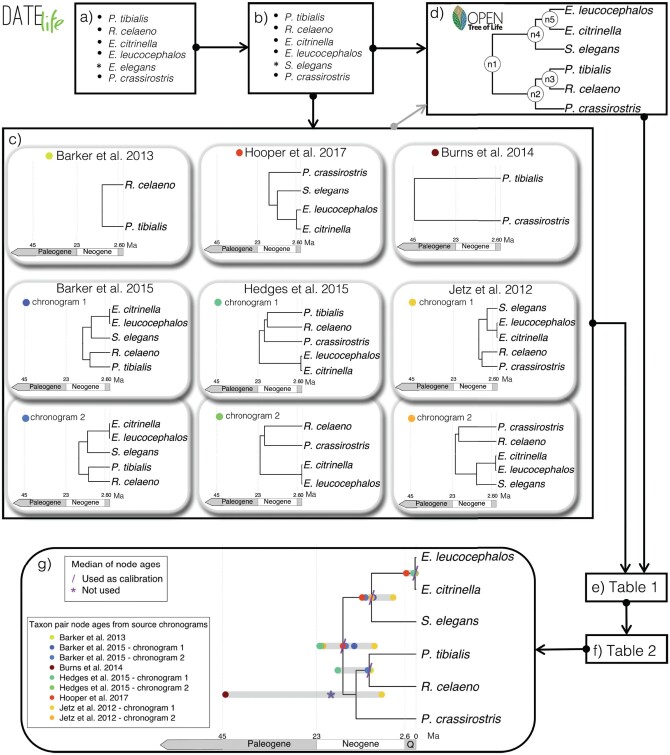
DateLife results of an analysis of a small sample of 6 bird species within the Passeriformes. Input species names (a) were processed (b) and were found across 9 chronograms within 6 independent studies (c; Barker et al. (2012), Barker et al. (2015), Burns et al. (2014), Hedges et al. (2015), Hooper and Price (2017), Jetz et al. (2012). This revealed 28 source age data points for the queried species names (e; Table 1). Summarized age data (f; Table 2) was used as secondary calibrations to date a tree topology obtained from OpenTree’s synthetic tree v13.4 (d), resulting in the chronogram of summary source ages shown in (g). The Paleogene Period spans from the end of the Cretaceous Period 66 million years ago (Ma) to the beginning of the Neogene Period 23.03 Ma. Q = Quaternary period.

**Figure 3:**
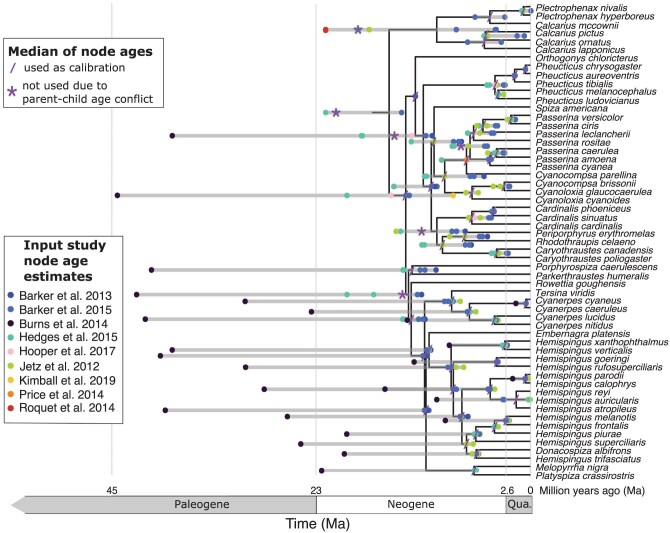
Median summary chronogram resulting from a DateLife analysis of bird species within the family Fringillidae. For visualization purposes, we are showing a portion of the final median summary chronogram encompassing 57 species out of the 289 total included in the analysis. The complete final chronogram is available as Supplementary Figure S3. The starting tree topology (Supplementary Fig. S2) has 289 tip species and 253 nodes; DateLife revealed age data for 194 of these nodes from at least one published chronogram. In total, 19 different chronograms from 13 different studies contributed 818 age data points, which were summarized to obtain a single value for each one of the 194 nodes with age data. From the 194 summary ages available, 21 were discarded and not used as calibrations (asterisk, *), because they were older than a parent node or younger than a descendant node; the remaining 173 summary ages were used as secondary calibrations (forward slash, /) with the Branch Length Adjuster (BLADJ) software from Webb et al., (2008). The Paleogene Period spans from the end of the Cretaceous Period 66 million years ago (Ma) to the beginning of the Neogene Period 23.03 Ma. Qua. = Quaternary period.

We illustrate the DateLife workflow using a family within the passeriform birds encompassing the true finches, Fringillidae, as case study. On a small example, we analysed 6 bird species, and results from each step of the workflow are shown in Figure 2. As a second example, we analysed 289 bird species in the family Fringillidae that are included in the NCBI taxonomy. One clade from the full summary chronogram result from the DateLife analysis is shown in Figure 3. The full chronogram for all 289 species and the results from previous steps of the workflow are available in Supplementary Figures.

### A Small Example

#### Creating a search query.—

We chose 6 bird species within the Passeriformes. The sample includes 2 species of cardinals: the black-thighed grosbeak—*Pheucticus tibialis* and the crimson-collared grosbeak—*Rhodothraupis celaeno*; three species of buntings: the yellowhammer—*Emberiza citrinella*, the pine bunting—*Emberiza leucocephalos* and the yellow-throated bunting—*Emberiza elegans*; and one species of tanager, the vegetarian finch—*Platyspiza crassirostris*. Processing of input names found that *Emberiza elegans* is synonym for *Schoeniclus elegans* in the default reference taxonomy (OTT v3.3, June 1, 2021). For a detailed discussion on the state of the synonym, refer to Avibase (Avibase 2022; Lepage 2023; Lepage et al. 2014). Discovering this synonym allowed assigning 5 age data points for the parent node of *Emberiza elegans*, shown as *Schoeniclus elegans* in Figure 2, which would not have had any data otherwise.

#### Searching the database.—

DateLife used the processed input names to search the local chronogram database and found 9 matching chronograms from 6 different studies (Fig. 2c). Three studies matched 5 input names (Barker et al. 2015; Hedges et al. 2015; Jetz et al. 2012), one study matched 4 input names (Hooper & Price 2017), and 2 studies matched 2 input names (Barker et al. 2013; Burns et al. 2014). No studies matched all input names. Together, source chronograms provide 28 unique age data points, covering all nodes on our chosen tree topology to date (Table 1).

**Table 1:** Node ages extracted with DateLife from 9 chronograms (shown in Fig. 2c) containing at least 2 of the 6 bird species analysed in the Fringillidae small example. All nodes are congruent with the chosen tree topology shown in Figure 2d.

Node name	Taxon A	Taxon B	Node age	Study reference
n1	*Emberiza citrinella*	*Platyspiza crassirostris*	9.651	Jetz et al. (2012) - chronogram 1
n1	*Pheucticus tibialis*	*Schoeniclus elegans*	14.334	Barker et al. (2015) - chronogram 1
n1	*Rhodothraupis celaeno*	*Schoeniclus elegans*	16.298	Barker et al. (2015) - chronogram 2
n1	*Emberiza citrinella*	*Platyspiza crassirostris*	16.950	Hooper & Price (2017)
n1	*Rhodothraupis celaeno*	*Schoeniclus elegans*	21.514	Jetz et al. (2012) - chronogram 2
n1	*Emberiza leucocephalos*	*Platyspiza crassirostris*	22.000	Hedges et al. (2015) - chronogram 2
n1	*Emberiza citrinella*	*Platyspiza crassirostris*	22.376	Hedges et al. (2015) - chronogram 1
n2	*Platyspiza crassirostris*	*Rhodothraupis celaeno*	7.969	Jetz et al. (2012) - chronogram 1
n2	*Platyspiza crassirostris*	*Rhodothraupis celaeno*	19.709	Jetz et al. (2012) - chronogram 2
n2	*Platyspiza crassirostris*	*Rhodothraupis celaeno*	19.709	Hedges et al. (2015) - chronogram 2
n2	*Platyspiza crassirostris*	*Rhodothraupis celaeno*	19.713	Hedges et al. (2015) - chronogram 1
n2	*Pheucticus tibialis*	*Platyspiza crassirostris*	44.296	Burns et al. (2014)
n3	*Pheucticus tibialis*	*Rhodothraupis celaeno*	10.530	Barker et al. (2015) - chronogram 1
n3	*Pheucticus tibialis*	*Rhodothraupis celaeno*	10.538	Barker et al. (2013)
n3	*Pheucticus tibialis*	*Rhodothraupis celaeno*	11.210	Barker et al. (2015) - chronogram 2
n3	*Pheucticus tibialis*	*Rhodothraupis celaeno*	18.157	Hedges et al. (2015) - chronogram 1
n4	*Emberiza citrinella*	*Schoeniclus elegans*	5.324	Jetz et al. (2012) - chronogram 1
n4	*Emberiza leucocephalos*	*Schoeniclus elegans*	9.862	Barker et al. (2015) - chronogram 1
n4	*Emberiza leucocephalos*	*Schoeniclus elegans*	10.339	Jetz et al. (2012) - chronogram 2
n4	*Emberiza leucocephalos*	*Schoeniclus elegans*	11.732	Barker et al. (2015) - chronogram 2
n4	*Emberiza citrinella*	*Schoeniclus elegans*	12.513	Hooper & Price (2017)
n5	*Emberiza citrinella*	*Emberiza leucocephalos*	0.141	Jetz et al. (2012) - chronogram 1
n5	*Emberiza citrinella*	*Emberiza leucocephalos*	0.152	Hedges et al. (2015) - chronogram 2
n5	*Emberiza citrinella*	*Emberiza leucocephalos*	0.201	Barker et al. (2015) - chronogram 1
n5	*Emberiza citrinella*	*Emberiza leucocephalos*	0.241	Barker et al. (2015) - chronogram 2
n5	*Emberiza citrinella*	*Emberiza leucocephalos*	0.273	Jetz et al. (2012) - chronogram 2
n5	*Emberiza citrinella*	*Emberiza leucocephalos*	0.576	Hedges et al. (2015) - chronogram 1
n5	*Emberiza citrinella*	*Emberiza leucocephalos*	2.290	Hooper & Price (2017)

#### Summarizing search results.—

DateLife obtained OpenTree’s synthetic tree topology for these taxa (Fig. 2d), and congruified and mapped age data to nodes in this chosen topology, shown in Table 1. The name processing step allowed including 5 data points for node “n4” (parent of *Schoeniclus elegans*) that would not have had any data otherwise due to taxonomic name mismatch. Age summary statistics per node were calculated (Table 2) and used as calibrations to date the tree topology using the BLADJ algorithm. As expected, more inclusive nodes (e.g., node “n1”) have more variance in age data than less inclusive nodes (e.g., node “n5”). Median summary age data for node “n2” was excluded as final calibration because it is older than the median age of a more inclusive node, “n1” (Fig. 2g).

**Table 2:** Summary statistics of node ages from Table 1

Node name	Min age	Q1	Median age	Mean age	Q3	Max age	Variance	SD
n1	9.651	15.316	16.950	17.589	21.757	22.376	22.243	4.716
n2	7.969	19.709	19.709	22.279	19.713	44.296	177.328	13.316
n3	10.530	10.536	10.874	12.609	12.946	18.157	13.783	3.713
n4	5.324	9.862	10.339	9.954	11.732	12.513	7.826	2.798
n5	0.141	0.176	0.241	0.553	0.425	2.290	0.608	0.780

Note: DateLife uses median ages by default to date a chosen tree topology (Fig. 2g).

### An Example with the Family of True Finches

#### Creating a query.—

To obtain ages for all species within the family of true finches, Fringillidae, we ran a DateLife query using the “get species from taxon” flag, which gets all recognized species names within a named group from a taxonomy of choice. Following the NCBI taxonomy, our DateLife query has 289 Fringillidae species names. This taxon-constrained approach implies that the full DateLife analysis will be performed using a tree topology and ages available for species names from a given taxonomic group, which do not necessarily correspond to a monophyletic group. Users can change this behavior by providing all species names corresponding to a monophyletic group as input for a DateLife search, or a monophyletic tree to construct a DateLife summary.

#### Searching the database.—

Next, we used the processed species names in our DateLife query to identify chronograms with at least 2 Fringillidae species as tip taxa. The DateLife search identified 19 chronograms matching this criteria, published in 13 different studies (Barker et al. 2013, 2015; Burns et al. 2014; Claramunt & Cracraft 2015; Gibb et al. 2015; Hedges et al. 2015; Hooper & Price 2017; Jetz et al. 2012; Kimball et al. 2019; Oliveros et al. 2019; Price et al. 2014; Roquet et al. 2014; Uyeda et al. 2017). Once identified, DateLife pruned these matching chronograms to remove tips that do not belong to the queried taxon names, and transformed these pruned chronograms to pairwise distance matrices, revealing 1,206 different age data points available for species within the Fringillidae (Supplementary Table S1).

#### Summarizing search results.—

The final step entailed congruifying and summarizing the age data available for the Fringillidae species into 2 single summary chronograms, using 2 different types of summary ages, median and SDM. As explained in the “Description” section, a tree topology to summarize age data upon is required. By default, DateLife uses the topology from OpenTree’s synthetic tree that contains all taxa from the search query. According to OpenTree’s synthetic tree, species belonging to the family Fringillidae do not form a monophyletic group (Supplementary Fig. S1). Hence, a topology containing only the 289 species from the original query was extracted from Open Tree of Life’s synthetic tree v12.3 (Supplementary Fig. S2; Open Tree Of Life et al. 2019).

All 19 source chronograms (Supplementary Figs. S5–S23) were congruified to OpenTree’s topology shown in Supplementary Figure S2, reducing the original 1,206 node age data set to 818 different data points (Supplementary Table S2) that could be used as calibrations for that chosen topology. The congruent node age data points were summarized for each node, resulting in 194 summary node ages. From these, 21 were excluded as secondary calibrations because they were older than the ancestral node. The remaining 173 summary node ages were used as secondary calibrations to obtain a fully dated (and resolved) phylogeny with the program BLADJ (Supplementary Fig. S3). Results for a subgroup are shown in Figure 3.

## Cross-Validation Test

**Figure 4:**
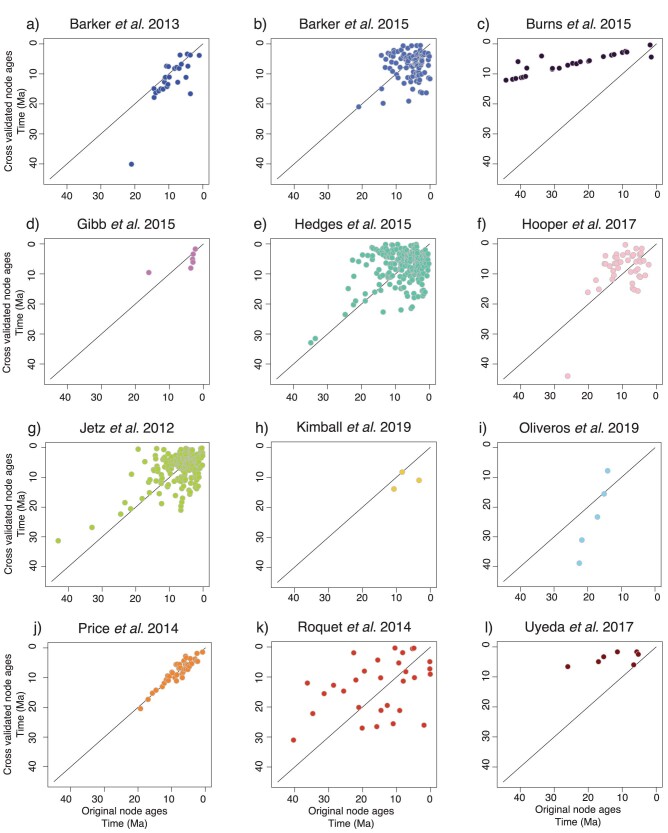
Cross validation of results from a DateLife analysis of the family Fringillidae, shown in Figure 3 and Supplementary Figure S3. Each plot compares the original node age estimates from an input source study chronogram (*x*-axis) with the corresponding cross-validated node age resulting from a dating analysis using the DateLife workflow, excluding data from that study (*y*-axis). It follows that points below the identity line (y=x) represent nodes with ages that are younger in the original study, and points above represent nodes that are older in the original study, compared to cross-validated ages.

We performed a cross-validation test of a DateLife analysis using the Fringillidae source chronograms obtained above (Supplementary Figs. S5–S23). As inputs for a DateLife analysis, we used all individual tree topologies from each of the 19 source chronograms from 13 studies, treating their node ages as unknown. We congruified node ages extracted from chronograms from all other studies upon the individual topologies, effectively excluding original ages from each topology. Finally, average node ages per node were applied as secondary calibrations and smoothed with the BLADJ algorithm. We found that node ages from the original studies, and ages estimated using all other age data available are generally correlated (Fig. 4). For five studies, DateLife tended to underestimate ages for topologically deeper nodes (those with many descendant taxa, aka “closer to the root”) relative to the original estimate, and overestimate ages for nodes closer to the tips. Accordingly, root ages are generally older in the original study than estimated using cross-validated ages (Supplementary Fig. S4). In general, topologically deeper nodes display the largest age variation between node ages from the original chronograms and ages summarized with DateLife.

## Discussion

DateLife’s goal is to improve availability, accessibility, and reusability of state-of-the-art data on evolutionary time frame of organisms, to allow users from all areas of science and with all levels of expertise to compare, use and reanalyse expert age data for their own applications. As such, it is designed as an open service that does not require any expert biological knowledge—besides the scientific names of the species or group that users want to work with—to use any of its functionalities.

A total of 99,474 unique terminal taxa are represented in DateLife’s database. Incorporation of more chronograms into the database will continue to improve DateLife’s services. One option to increase the number of chronograms in the DateLife database is the Dryad data repository. Methods to automatically mine chronograms from Dryad could be designed and implemented. However, Dryad’s metadata system has no information to automatically detect branch length units, and those would still need to be determined manually by a human curator. We would like to emphasize on the importance of sharing chronogram data, including systematically curated metadata, into open repositories, such as OpenTree’s Phylesystem (McTavish et al. 2015) for the benefit of research and the scientific community as a whole. Another important source of expert data on time of lineage divergence is TimeTree’s database (Hedges et al. 2006), which holds chronograms from more than 4k published studies, and is fully browsable using its graphical user interface (timetree.org). TimeTree’s chronogram database was not accessible in computer readable format until very recently (Kumar et al. 2022), when its terms of use and website application were updated, now allowing some kinds of reuse, but not redistribution.

The inaccessibility of TimeTree’s database was an inspiration for the DateLife project, which was born as a prototype tool initially developed over a series of hackathons at the National Evolutionary Synthesis Center, NC, USA (Stoltzfus et al. 2013), as the need to make scientific information that is funded by the public practically available to the public was acknowledged and prioritized.

As we envision that DateLife will have many interesting applications in research and beyond, we emphasize that DateLife’s results—as well as any insights gleaned from them, largely depend on the quality of the source chronograms: low quality chronograms will produce low-quality results. The “garbage in, garbage out” problem has long been recognised in supertree methods for summarizing phylogenetic trees (Bininda-Emonds et al. 2004). We note that this is a surfacing issue of any automated tool for biological data analysis. For example, DNA riddled with sequencing errors will produce generally poor alignments that will return biased evolutionary hypothesis, independently of the quality of the analysis software used. Again, we urge readers and DateLife users to explore all input chronograms before using a summary chronogram resulting from a DateLife workflow.

Finally, uncertainty and variability of chronogram node age estimates might pose larger issues in some research areas than others. For example, in ecological and conservation biology studies, it has been shown that incorporating some chronogram data provides better results than when not using any age data at all, even if the node ages are not the best quality (Webb et al. 2008). In the following sections, we discuss the particularities of divergence times from DateLife’s summary chronograms and their impact on certain evolutionary analyses, for consideration of the readers and users in different research areas.

### Age Variation in Source Chronograms

Conflict in estimated ages among alternative studies is common in the literature. See, for example, the robust ongoing debate about crown group age of angiosperms (Barba-Montoya et al. 2018; Magallón et al. 2015; Ramshaw et al. 1972; Sanderson & Doyle 2001; Sauquet et al. 2021). Alternative source chronograms available for the same taxa have potentially been estimated implementing different types of calibrations, which affects the resulting node age estimates. For example, in the DateLife analysis of the Fringillidae shown above, the chronograms from one study (Burns et al. 2014) were inferred using molecular substitution rate estimates across birds (Weir & Schluter 2008), and have much older age estimates for the same nodes than chronograms that were inferred using fossil calibrations (Figs. 3, 4c; Supplementary Figs. S4c, S10). Another source of conflict in estimated node ages can arise from different placements for the same calibration, which would imply fundamentally distinct evolutionary hypotheses (Antonelli et al. 2017). For example, two independent researchers working on the same clade should both carefully select and justify their choices of fossil calibration placement. Yet, if one researcher concludes that a fossil should calibrate the ingroup of a clade, while another researcher concludes that the same fossil should calibrate the outgroup of the clade, the resulting age estimates will differ, as the placement of calibrations as stem or crown group is known to significantly affect estimates of time of lineage divergence (Sauquet 2013). Finally, placement of calibrations also affects uncertainty of node age estimates. For example, nodes that are sandwiched between a calibrated node and a calibrated root have less freedom of movement and hence narrower confidence intervals (Vos & Mooers 2004), which inflates precision for nodes without calibrations but does not necessarily improve accuracy of the estimated ages.

DateLife’s summary chronograms are intended to represent all variation in estimated node ages from source chronograms. Node age distribution ranges allow to visually explore ages from source chronograms individually and contextualize and compare them against other chronograms. Researchers that wish to use summary chronograms in downstream evolutionary analysis may select multiple trees sampled from the summary distribution of node ages, to account for variation in source chronograms.

### Primary vs Secondary Calibrations

DateLife constructs summary chronograms using node ages extracted from existing chronograms, i.e., secondary calibrations. In general, the scientific community has more confidence in chronograms using primary calibrations, where the dated tree is generated from a single analysis where carefully chosen fossil calibrations are the source of absolute time information, than in analyses dated using secondary calibrations (Antonelli et al. 2017; Garzón-Orduña et al. 2015; Graur & Martin 2004; Sauquet 2013; Sauquet et al. 2012; Schenk 2016; Shaul & Graur 2002). However, implementation of primary calibrations is difficult: it requires specialized expertise and training to discover, place and apply calibrations appropriately (Hipsley & Müller 2014; Ksepka et al. 2011). One approach is to use fossils that have been widely discussed and previously curated as calibrations to date other trees (Ksepka et al. 2011; Sauquet 2013), and making sure that all data reflect a coherent evolutionary history (Sauquet 2013), as for example done by Antonelli et al. (2017). The Fossil Calibration Database provides data for 220 primary calibration points encompassing flowering plants and metazoans, that have been curated by experts and used for dating analysis in peer-reviewed publications (Ksepka et al. 2015). This database facilitates the use of expert primary fossil calibrations in new phylogenetic dating analyses. Yet, users still require the expertise to locate and calibrate appropriate nodes in their phylogenies which correspond with fossils available in the database.

Recently, Powell et al. (2020) showed in a simulation study that secondary calibrations using node ages based on previous molecular clock analyses can be as good as primary calibrations. Using several secondary calibrations (as opposed to just one) can provide sufficient information to alleviate or even neutralize potential biases (Graur & Martin 2004; Sauquet 2013; Shaul & Graur 2002). Our cross-validation analysis also provides insight into the application of secondary calibrations. Node ages summarized with DateLife and those from the original studies are well correlated (Supplementary Figs. S5–S23). We also note that DateLife estimates for nodes closer to the root tend to be slightly younger than ages from the original studies. In contrast, nodes closer to the tips tend to be slightly older when estimated using our secondary calibrations than ages from the original studies. The only exception to this trend was observed by Burns et al. (2014) chronogram, which generally displays much younger node ages when estimated using secondary calibrations than the original study (Supplementary Figs. S4c, S10), supporting previous observations (Sauquet et al. 2012; Schenk 2016). However, these younger dates are more likely an example of how multiple secondary calibrations can correct erroneous estimates, as dates on the Burns et al. (2014) tree were obtained using a single secondary calibration based on a previously estimated molecular evolution rate across birds from Weir & Schluter (2008), and appear as major outliers compared to alternate estimates for the same nodes based on primary fossil calibrations (Fig. 3; Supplementary Fig. S3).

### Sumarizing Chronograms

By default, DateLife currently summarizes all source chronograms that overlap with at least 2 species names. Users can exclude source chronograms if they have reasons to do so. Strictly speaking, a good chronogram should reflect the real time of lineage divergence accurately and precisely. To our knowledge, there are no tested measures to determine independently if a chronogram is better than another. Yet, several characteristics of the data used for dating analyses, as well as from the output chronogram itself, could be used to score the quality of source chronograms.

Some measures that have been proposed are the proportion of lineage sampling and the number of calibrations used (Magallón 2010; Magallón et al. 2015). Some characteristics that are often cited in published studies as a measure of improved age estimates as compared to previously published estimates are: quality of alignment (missing data, GC content), lineage sampling (strategy and proportion), phylogenetic and dating inference method, number of fossils used as calibrations, support for nodes and ages, and magnitude of confidence intervals.

DateLife provides an opportunity to capture concordance and conflict among date estimates, which can also be used as a metric for chronogram reliability. Its open database of chronograms allows other researchers to do such analyses themselves reproducibly, and without needing permission. Though, of course, they should follow proper citation practices, especially for the source chronogram studies.

The exercise of summarizing age data from across multiple studies is a common resource in research, as it provides the opportunity to work with a chronogram that reflects a unified evolutionary history for a lineage, by putting together evidence from different hypotheses. For example, the largest, and taxonomically broadest chronogram currently available from OpenTree was constructed summarizing age data from 2,274 published chronograms using NCBI’s taxonomic tree as backbone (Hedges et al. 2015), which has been widely reused for research.

Finally, we note that summarizing chronograms should be done with caution, as it may amplify the effect of uncertainty and errors in source data, and blur parts of the evolutionary history of a lineage that might only be reflected in source chronograms and lost on the summary chronogram (Sauquet et al. 2021).

### Effects of Taxon Sampling on Downstream Analyses

Analysis of species diversification of simulated and empirical phylogenies suggest that using a more completely sampled phylogeny provides estimates that are closer to the true diversification history than when analysing incompletely sampled phylogenies (Chang et al. 2020; Cusimano et al. 2012; Sun et al. 2020). Ideally, phylogenies should be completed using genetic data, but this is a time-consuming and difficult task to achieve for many biological groups. Hence, DateLife’s workflow features different ways of assigning divergence times to taxa in the absence of direct information from calibrations and branch lengths.

Completing a phylogeny using a stochastic birth–death polytomy resolver and a backbone taxonomy is a common practice in scientific publications: Jetz et al. (2012), created a chronogram of all 9993 bird species, where 67% had molecular data and the rest was simulated; Rabosky et al. (2018) created a chronogram of 31,536 ray-finned fishes, of which only 37% had molecular data; Smith (2018) constructed a chronogram of 353,185 seed plants where only 23% had molecular data. Stochastically resolved chronograms can return diversification rates estimates that appear less biased than those estimated from their incompletely sampled counterparts, even with methods that account for missing lineages by using sampling fractions (Chang et al. 2020; Cusimano et al. 2012) but can also introduce spurious patterns of early bursts of diversification (Cusimano et al. 2012; Sun et al. 2020).

Taxonomy-based stochastic polytomy resolvers also introduce topological differences in phylogenetic trees. The study of macroevolutionary processes largely depends on an understanding of the timing of species diversification events, and different phylogenetic and chronogram hypotheses can provide very different overviews of the macroevolutionary history of a biological group. For example, alternative topologies in chronograms from the same biological group can infer very different species diversification patterns (Rabosky 2015; Title & Rabosk 2016). Similarly, there are worries that patterns of morphological evolution cannot be accurately inferred with phylogenies that have been resolved stochastically over a taxonomic backbone, as any patterns would be erased by randomization (Rabosky 2015). We note that the same applies for geography- and morphology-dependent diversification analyses. Hence, we suggest that phylogenies that have been processed with taxonomy-based stochastic polytomy resolvers, including certain summary chronograms from a DateLife analysis, can be useful as null or neutral models, representing the case of a diversification process that is independent of traits and geographical scenario.

Taxonomy-based stochastic polytomy resolvers have been used to advance research in evolution, still, risks come with this practice. Taken to the extreme, one could generate a fully resolved, calibrated tree of all modern and extinct taxa using a single taxonomy, a single calibration, and assigning branch lengths following a birth–death diversification model. Clearly, this can lead to a misrepresentation of the true evolutionary history. We urge DateLife users to follow the example of the large tree papers cited above, by carefully considering the statistical assumptions being made, potential biases, and assessing the consistency of DateLife’s results with prior work.

## Conclusions

Knowledge of the evolutionary time frame of organisms is key to many research areas: trait evolution, species diversification, biogeography, macroecology, and more. It is also crucial for education, science communication and policy, but generating chronograms is difficult, especially for those who want to use phylogenies but who are not systematists, or do not have the time to acquire and develop the necessary knowledge and skills to construct chronograms on their own. Importantly, years of primarily public-funded research have resulted in vast amounts of chronograms that are already available in scientific publications, but functionally hidden from the public and scientific community for reuse.

The DateLife project allows for easy and fast summarization of public and state-of-the-art data on time of lineage divergence. It is available as an R package, and as a web-based R shiny application at www.datelife.org. DateLife provides a straightforward way to get an informed picture of the state of knowledge for the time frame of evolution of different regions of the tree of life and allows identifying regions that require more research, or that have conflicting information. Additionally, both summary and newly generated trees using the DateLife workflow are useful to evaluate evolutionary hypotheses in different areas of research. We hope that the DateLife project will increase awareness of the existing variation in expert estimations of time of divergence, and foster exploration of the effect of alternative divergence time hypotheses on the results of analyses, nurturing a culture of more cautious interpretation of evolutionary results.

## Data Availability

The DateLife software is free and open source. It can be used online through its R shiny web application hosted at http://www.datelife.org, and locally through the datelife R package, available from Zenodo (https://doi.org/10.5281/zenodo.593938 and the CRAN repository (Sanchez-Reyes et al. 2022). DateLife’s web application is maintained using RStudio’s shiny server and the shiny package open infrastructure, as well as Docker and OpenTree’s infrastructure (datelife.opentreeoflife.org). datelife’s stable version can be installed from the CRAN repository using the command install.packages(pkgs = "datelife") from within R. Development versions are available from DateLife’s GitHub repository (https://github.com/phylotastic/datelife) and can be installed using the command dev-tools::install_github("phylotastic/datelife").

## Supplementary Material

Supplementary material, including figures, tables, code, biological examples, benchmark results, data files, and online-only appendices, can be viewed and downloaded from the Dryad data repository (https://doi.org/10.5061/dryad.cnp5hqc6w), as well as from the Zenodo stable repositories that host the reproducible manuscript (https://doi.org/10.5281/zenodo.7435094), the software benchmark (https://doi.org/10.5281/zenodo.7435101), the biological examples (https://doi.org/10.5281/zenodo.7435106), and the figures (https://doi.org/10.5281/zenodo.6683667). Dryad’s data publication fee is covered by the Society of Systematic Biologists. Development versions corresponding to all of the above are hosted on GitHub, accessible at https://github.com/LunaSare/datelifeMS1, https://github.com/LunaSare/datelife_examples, and https://github.com/LunaSare/datelife_benchmark.
